# Genome Sequence of *Ralstonia pseudosolanacearum* Strains with Compatible and Incompatible Interactions with the Major Tomato Resistance Source Hawaii 7996

**DOI:** 10.1128/genomeA.00982-17

**Published:** 2017-09-07

**Authors:** Greecy M. R. Albuquerque, Elineide B. Souza, Adriano M. F. Silva, Carlos A. Lopes, Leonardo S. Boiteux, Maria Esther de N. Fonseca

**Affiliations:** a Department of Agronomy, Universidade Federal Rural de Pernambuco, Recife, Pernambuco, Brazil; b Department of Biology, Universidade Federal Rural de Pernambuco, Recife, Pernambuco, Brazil; c National Center for Vegetable Crops Research (CNPH), Embrapa Hortaliças, Brasília, Federal District, Brazil

## Abstract

We report here the complete genome sequences of two *Ralstonia pseudosolanacearum* strains, isolated from the warm northeast region of Brazil. They display divergent (compatible versus incompatible) interactions with the resistant tomato line Hawaii 7996. Polymorphisms were detected in a subset of effector genes that might be associated with these contrasting phenotypes.

## GENOME ANNOUNCEMENT

*Ralstonia pseudosolanacearum* is a soilborne pathogen and one of the main causal agents of the bacterial wilt (BW) disease of tomato (*Solanum lycopersicum* L.) and other crops (1). *R. pseudosolanacearum* is currently classified as a distinct species within the *R. solanacearum* complex, which comprises strains of phylotypes I and III (2–5). Although they are of putative exotic origin, *R. pseudosolanacearum* phylotype I isolates are currently disseminated in Brazil (north, northeast, and central regions) and infect mainly *Solanaceae* crops (tomato, peppers, eggplant, and scarlet eggplant) (6, 7).

In this study, two tomato-infecting *R. pseudosolanacearum* strains from the warm Brazilian northeast region were sequenced in order to analyze candidate genes associated with their divergent (compatible versus incompatible) interactions with the tomato line Hawaii 7996, which is the main breeding source of BW resistance in this vegetable crop (8, 9). Strain RS 476 (sequevar I-18 from Maranhão state) is characterized by its ability to induce severe BW symptoms on Hawaii 7996 (≈60% incidence), whereas strain CRMRs218 (also sequevar I-18 from Pernambuco state) is able to induce severe BW symptoms in a wide range of tomato cultivars, but it is avirulent to Hawaii 7996.

The genomic DNA was extracted using the DNeasy blood and tissue kit (Qiagen, Hilden, Germany). The purified DNA quantification and quality analyses were done in a NanoDrop spectrophotometer (Thermo Scientific, Waltham, MA, USA) and in a fluorometer. The two genomic samples were sequenced on one lane of a HiSeq 2500 instrument (Illumina) at the Centro de Biotecnologia Animal (Esalq/Usp, São Paulo, SP, Brazil), and libraries were prepared with the Nextera DNA sample prep kit (Illumina, San Diego, CA, USA). Sequencing was performed with 100-bp paired-end reads, with genome coverages of ≈810× (RS 476) and ≈684× (CRMRs218). Quality control was done using FastQC (https://www.bioinformatics.babraham.ac.uk/projects/fastqc) in order to check Phred scores of the reads.

SeqMan NGen version 14 software (Lasergene, DNASTAR, Madison, WI, USA) was employed for genome assembly using *R. pseudosolanacearum* GMI1000 as the reference strain (GenBank assembly accession number GCA_000009125.1). The complete genomes of the two contrasting Brazilian strains were annotated in the NCBI Prokaryotic Annotation Pipeline (https://www.ncbi.nlm.nih.gov/genome/annotation_prok). Each consisted of two replicons, circular chromosomes of 3,716,855 bp for RS 476 and 3,720,116 bp for CRMRs218 and megaplasmids of 2,094,715 bp for RS 476 and 2,095,997 bp for CRMRs218.

The annotated genomes of RS 476 and CRMRs218 consisted, respectively, of 5,206 and 5,216 genes, 5,133 and 5,144 coding sequences, 5,000 and 4,882 protein-coding genes, 133 and 262 pseudogenes, 4 and 3 noncoding RNAs, and GC contents of 66.95% and 66.71%. These two strains presented three structural RNAs (5S, 16S, and 23S) and 57 tRNAs. Mutations (single-nucleotide polymorphisms and indels) were detected in a subset of effector genes that might be associated with these contrasting phenotypes, which will demand additional comparative genomic studies.

### Accession number(s).

The genome sequences (chromosomes and megaplasmids) reported here have been deposited in NCBI GenBank under the accession numbers CP021762 and CP021763 for the chromosome and plasmid, respectively, of strain RS 476 and CP021764 and CP021765 for the chromosome and plasmid, respectively, of strain CRMRs218.
